# Risk Factors for Syncope Associated With Multigenerational Relatives With a History of Syncope

**DOI:** 10.1001/jamanetworkopen.2021.2521

**Published:** 2021-03-30

**Authors:** Artur Fedorowski, Mirnabi Pirouzifard, Jan Sundquist, Kristina Sundquist, Richard Sutton, Bengt Zöller

**Affiliations:** 1 Department of Clinical Sciences, Lund University, Malmö, Sweden; 2 Department of Cardiology, Skåne University Hospital, Malmö, Sweden; 3 Center for Primary Health Care Research, Lund University/Region Skåne, Malmö, Sweden; 4 Stanford Prevention Research Center, Stanford University School of Medicine, Palo Alto, California; 5 National Heart and Lung Institute, Imperial College, Department of Cardiology, London, United Kingdom

## Abstract

**Importance:**

Reflex syncope is the major cause of transient loss of consciousness, which affects one-third of the population, but effective treatment for individuals with severe syncope is lacking. Better understanding of reflex syncope predisposition may offer new therapeutic solutions.

**Objectives:**

To determine the familial risk of syncope in first-, second-, and third-degree relatives of affected individuals and to explore the role of genes and family environment in reflex syncope.

**Design, Setting, and Participants:**

In this national population-based family cohort study, the Swedish multigeneration register was linked to 3 Swedish nationwide registers: hospital discharge, outpatient care, and primary care registers for the period from 1997 to 2015. Sibling pairs born to Swedish parents between 1948 and 2005 were included. Linkage was also made to half-siblings and cousins. Data analysis was performed from June to October 2020.

**Exposures:**

Register-based syncope diagnosis among relatives: pairs of twins, siblings, half-siblings, and cousins.

**Main Outcomes and Measures:**

Odds ratios for syncope were calculated for relatives (twins, siblings, half-siblings, and cousins) of individuals who had syncope compared with relatives of individuals without syncope for reference. Sensitivity analysis excluding families with definite nonreflex syncope diagnosis was performed.

**Results:**

Among the study population of 2 694 442 participants, 1 381 453 (51.3%) were male, and the median (interquartile range) age was 32 (22-43) years. The study population consisted of 24 020 twins, 1 546 108 siblings, 264 244 half-siblings, and 1 044 546 cousins. In total, 61 861 (2.30%) unique individuals were diagnosed with syncope. Sixty-two percent (38 226) of the syncope-positive individuals were female. The odds ratio (OR) for syncope was 2.39 (95% CI, 1.61-3.53) for twins, 1.81 (95% CI, 1.71-1.91) for siblings, 1.28 (95% CI, 1.20-1.37) for half-siblings, and 1.13 (95% CI, 1.10-1.17) for cousins of individuals with syncope. The OR was highest among male twins at 5.03 (95% CI, 2.57-9.85). The proportion of syncope-positive individuals was consistently higher in women vs men, regardless of degree of relationship (twins: 346 [2.88%] vs 193 [1.61%]; siblings: 22 111 [2.92%] vs 13 419 [1.70%], half-siblings: 4148 [3.44%] vs 2425 [1.93%], cousins: 14 498 [2.87%] vs 9246 [1.72%]). Exclusion of nonreflex syncope diagnoses did not change syncope risk in affected families.

**Conclusions and Relevance:**

In this Swedish national population-based study, the risk of syncope among relatives of affected individuals was associated with the relationship degree and was strongest in twins and siblings, which suggests that there are genetic components of reflex syncope. Women were more likely to experience syncope independently of family relationship. A better understanding of genetic predisposition to reflex syncope may offer new therapeutic options in severely affected individuals.

## Key Points

**Question:**

Is there a familial risk of syncope in first-, second-, and third-degree relatives of individuals who have had syncope?

**Findings:**

In this population-based cohort study among adults in Sweden, family history of syncope was associated with increased incidence of syncope, and the familial risk was associated with genetic resemblance and age.

**Meaning:**

These findings suggest that syncope aggregates in families, which indicates that there may be genetic components of reflex syncope.

## Teaser

This cohort study examines whether a family history of syncope among first-, second-, or third-degree relatives is associated with an increased risk for syncope.

## Introduction

Reflex syncope is the most common form of transient loss of consciousness (TLOC), defined as sudden-onset bradycardia and vasodilation leading to an abrupt decrease in cardiac output, cerebral hypoperfusion, and temporary loss of consciousness, typically triggered by orthostatic or emotional stress, pain, and gastrointestinal symptoms.^1,2^ According to self-reported epidemiological data, approximately one-third of the human population may be affected by reflex syncope during their lifetime.^3^ Although prognosis of reflex syncope is usually benign, especially in younger individuals, recent observations have indicated that admission for syncope in middle and advanced age heralds an increased risk of future cardiovascular events and higher mortality.^4,5^ Moreover, syncope is associated with trauma risk in affected individuals,^6^ whereas reflex syncope at a young age may be correlated with family history of cardiovascular disease^7^ and coronary events many years later.^8^

Why some individuals are susceptible to the reflex syncope, often referred to as vasovagal,^1^ is not well understood. Studies on familial predisposition to reflex syncope have suggested higher incidence in affected families, especially among twins and for episodes before age 20 years.^9,10,11,12,13^ However, candidate gene association studies on reflex syncope have been inconclusive, difficult to replicate, and generally based on small patient series, which precludes generalizability.^14^ Recently, Sheldon et al^15^ have reported gender-specific association of single-nucleotide variation in genes involved in serotonin signaling with reflex syncope likelihood, and a genome-wide association study has identified single-nucleotide variation in proximity to the gene zinc finger protein 804a that is associated with vasovagal syncope.^16^ Even if these reports suggest heredity, large population-based studies on familial aggregation of reflex syncope are lacking.

Consequently, the aim of this study was to determine the familial risk of syncope among first-, second-, and third-degree relatives by linking several Swedish nationwide registers including the multigeneration family register.

## Methods

### Study Population

All data were provided by Statistics Sweden and the National Board of Health and Welfare for research purposes. Data were coded according to European Union law. The Regional Ethical Review Board in Lund, Sweden, approved this cohort study and waived informed consent as a requirement. This study followed the Strengthening the Reporting of Observational Studies in Epidemiology (STROBE) reporting guideline. We used the following Swedish national registers for data extraction: the Swedish Multi-Generation Register, which contains data on familial relationships and index persons born in 1932 and later and registered in Sweden 1961 and later^17^; the National Patient Register, which includes all hospital discharge diagnoses from 1964 to 2015 and nationwide coverage from 1987, and hospital outpatient diagnoses from 2001 to 2015; the Primary Healthcare Register, for 1989 to 2016; the national statistical register, which contains data on death date, if applicable, name change, marital status, family relationships, education, and migration (the register has high coverage for nearly 100% of birth and death dates, 95% of immigration events, and 91% for emigration events)^18^; and the Swedish Cause of Death Register, which provides date and cause of death from 1961 to 2015. The databases were linked together according to previously applied methods.^19^ As the specific diagnostic coding for reflex syncope is not available, the general code of unspecified syncope (R559; *International Statistical Classification of Diseases and Related Health Problems, Tenth Revision (ICD-10)* as a proxy of reflex syncope has been applied. We identified individuals with a diagnosis of syncope (*ICD-10* code R559) registered between 1997 and 2012 from the National Patient Register and between 1997 and 2015 from the Primary Healthcare Register. We had no access to the *ICD-10* syncope diagnosis code R559 from the National Patient register between 2013 and 2015.

In the Multi-generation National Swedish Register, we searched for families with 2 full biological siblings. Pairs of children born between 1948 and 2005 to parents born between 1932 and 1985 in Sweden were retrieved. The families with members who died or emigrated before 1997 or emigrated before the age of 17 years were excluded. Both biological parents were obligatorily known. Siblings were then linked with related families and 2 additional data sets for siblings’ half-siblings and cousins were also created. We applied the same criteria for related families as we did for siblings; that is, we excluded all half-siblings or cousins who were not born in Sweden, had emigrated before the age of 17, or had non–Swedish-born parents. Four different data sets were created: twins, siblings, half-siblings, and cousins.

Only full siblings but not twins and half-siblings were included in the sibling data set. In the database, all relative pairs were double-entered (ie, all sibling pairs, all twin pairs, all half-sibling pairs, and all cousin pairs, as described previously).^20^ We allowed the same person to be included in more than one family relationship.

### Statistical Analysis

Incidence rates were defined as the number of events divided by the person-time at risk. The familial incidence ratio between 2 incidence densities (rate in the exposed divided by rate in the unexposed) gave the incidence rate ratio. Syncope-free survival curves were constructed according to the Kaplan-Meier method to compare individuals with and without relative history of syncope. For comparison of 2 curves, the log-rank test, resulting in a test statistic with a χ^2^ distribution and 1 *df*, was used. Tetrachoric correlation was used to estimate correlation coefficients between relatives. The adjusted familial associations between twins’, siblings’, half siblings’, and cousins’ syncope events were further investigated with logistic regression. Results are reported as familial odds ratios (ORs) and 95% CIs. Models were adjusted for year of birth, sex, region at birth (county), and level of education. Familial ORs for syncope were calculated for relatives of individuals who had a diagnosis of syncope compared with relatives of individuals unaffected by syncope as the reference group. Stratified familial ORs were calculated according to sex and age. A sensitivity analysis was performed with exclusion of individuals with potential differential diagnosis (eTable 1 in the Supplement).

Pearson correlation was determined between genetic resemblance and tetrachoric correlation and familial ORs. To test the trend that there was a higher risk of syncope in relatives who were more closely related, we included all types of proband–relative pairs in a data set. The mean genetic resemblance for twins was determined with the Weinberg differential method. A total of 4139 twin pairs with different sex and 7871 twin pairs with same sex gave a mean genetic resemblance of 0.66 for twins (8278 dizygotic pairs and 3732 monozygotic pairs according to the Weinberg method). Each relative pair was assigned their genetic resemblance (ie, 0.66 for twin pairs, 0.5 for sibling-pairs, 0.25 for half-sibling pairs, and 0.125 for cousin pairs). We conducted the same logistic regression analysis as already described, but with inclusion of an interaction term between the genetic resemblance and syncope in relatives. Statistical significance was set at *P* < .05 and all tests were 2-tailed. Data were analyzed from June to October 2020 using SAS version 9.4 (SAS Institute).

## Results

Among the study’s 2 694 442 participants, 1 381 453 (51.3%) were male, and the median (interquartile range) age was 32 (22-43) years. The study population consisted of 1 570 128 siblings (of whom 24 020 were twins), 264 244 half-siblings, and 1 044 546 cousins. Table 1 summarizes the demographic characteristics of the study sample. A total of 61 861 participants (2.30%) were diagnosed with syncope during the period of 1997 to 2015. Of these, 29 762 (48.11%) were found in primary health care, 19 886 (32.15%) in the Outpatient Register, and 12 213 (19.74%) in the Hospital Discharge Register (Table 1). Of the syncope-positive individuals, 38 226 (62%) were female. Of the 24 020 twins, 539 (2.24%) had a diagnosis of syncope and 12 003 (49.97%) were female. Of these female twins, 346 (2.88%) had a syncope diagnosis. A higher proportion of female individuals than male individuals were affected by syncope in all relative pairs (twins: 346 [2.88%] vs 193 [1.61%]; siblings: 22 111 [2.92%] vs 13 419 [1.70%], half-siblings: 4148 [3.44%] vs 2425 [1.93%], cousins: 14 498 [2.87%] vs 9246 [1.72%]). The median (interquartile range) age of first syncope in the twins’ group was 17 (13-26) years. The median age of first syncope was slightly higher among other relative pairs (Table 1). The proportion of consanguinity in our data set was 0.2%.

**Table 1.** Characteristics of Study Population Stratified by the Degree of Family Relationship and Documented History of Syncope in Health Care Registers

Characteristics	Participants, No. (%)		
	All	Without syncope diagnosis	With syncope diagnosis
All			
Overall	2 694 442	2 632 581 (97.70)	61 861 (2.30)
Sex			
Male	1 381 453 (51.27)	1 357 818 (98.29)	23 635 (1.71)
Female	1 312 989 (48.73)	1 274 763 (97.09)	38 226 (2.91)
Year of birth, median (IQR) [range]	1983 (1972-1993) [1947-2005]	1983 (1972-1993) [1947-2005]	1986 (1974-1993) [1948-2005]
Age at end of follow-up, median (IQR) [range], y	32 (22-43) [0-68]	32 (22-43) [0-68]	29 (22-40) [9-67]
Age at syncope onset, median (IQR) [range], y	NA	NA	22 (16-33) [0-63]
Higher education (>11 y)	818 146 (30.36)	801 020 (97.91)	17 126 (2.09)
Source			
Primary care register	NA	NA	29 762 (48.11)
Hospital discharge register	NA	NA	12 213 (19.74)
Outpatient register	NA	NA	19 886 (32.15)
Twins			
Overall	24 020	23 481 (97.76)	539 (2.24)
Sex			
Male	12 017 (50.03)	11 824 (98.39)	193 (1.61)
Female	12 003 (49.97)	11 657 (97.12)	346 (2.88)
Year of birth, median (IQR) [range]	1992 (1981-1999) [1951-2005]	1992 (1981-1999) [1951-2005]	1991 (1982-1996) [1956-2005]
Age at end of follow-up, median (IQR) [range], y	22 (16-34) [0-64]	22 (16-34) [0-64]	24 (19-32) [10-59]
Age at syncope onset, median (IQR) [range], y	NA	NA	17 (13-26) [0-54]
Higher education (>11 y)	4940 (20.57)	4830 (97.77)	110 (2.23)
Source			
Primary health care register	NA	NA	274 (50.83)
Hospital discharge register	NA	NA	78 (14.47)
Outpatient register	NA	NA	187 (34.69)
Full siblings			
Overall	1 546 108	1 510 578 (97.70)	35 530 (2.30)
Sex			
Male	790 127 (51.10)	776 708 (98.30)	13 419 (1.70)
Female	755 981 (48.90)	733 870 (97.08)	22 111 (2.92)
Year of birth, median (IQR) [range]	1984 (1973-1993) [1948-2005]	1984 (1973-1993) [1948-2005]	1987 (1975-1993) [1951-2005]
Age at end of follow-up, median (IQR) [range], y	31 (21-42) [0-67]	31 (21-42) [0-67]	28 (22-40) [9-64]
Age at syncope onset, median (IQR) [range], y	NA	NA	21 (15-32) [0-63]
Higher education (>11 y)	476 280 (30.81)	466 320 (97.91)	9960 (2.09)
Source			
Primary care register	NA	NA	17 724 (49.88)
Hospital discharge register	NA	NA	6600 (18.58)
Outpatient register	NA	NA	11 209 (31.54)
Half-siblings			
Overall	246 244	239671 (97.33)	6573 (2.67)
Sex			
Male	125 670 (51.03)	123 245 (98.07)	2425 (1.93)
Female	1 205 574 (48.97)	116 426 (96.56)	4148 (3.44)
Year of birth, median (IQR) [range]	1984 (1973-1992) [1948-2005]	1984 (1973-1992) [1948-2005]	1986 (1976-1992) [1950-2005]
Age at end of follow-up, median (IQR) [range], y	31 (23-41) [0-67]	31 (23-41) [0-67]	29 (23-39) [10-65]
Age at syncope onset, median (IQR) [range], y	NA	NA	22 (16-32) [0-63]
Higher education (>11 y)	54 819 (22.26)	53 527 (97.64)	1292 (2.36)
Source			
Primary care register	NA	NA	2978 (45.31)
Hospital discharge register	NA	NA	1373 (20.89)
Outpatient register	NA	NA	2222 (33.80)
Cousins			
Overall	1 044 546	1 020 802 (97.73)	23 744 (2.27)
Sex			
Male	538 791 (51.58)	529 545 (98.28)	9246 (1.72)
Female	505 755 (48.42)	491 257 (97.13)	14 498 (2.87)
Year of birth, median (IQR) [range]	1982 (1971-1991) [1947-2005]	1982 (1971-1991) [1947-2005]	1984 (1973-1991) [1948-2005]
Age at end of follow-up, median (IQR) [range], y	33 (24-44) [0-68]	33 (24-44) [0-68]	30 (24-42) [10-67]
Age at syncope onset, median (IQR) [range], y	NA	NA	23 (16-34) [0-62]
Higher education (>11 y)	320 112 (30.65)	313 448 (97.92)	6664 (2.08)
Source			
Primary care register	NA	NA	10 913 (45.96)
Hospital discharge register	NA	NA	5069 (21.35)
Outpatient register	NA	NA	7762 (32.69)

Abbreviations: IQR, interquartile range; NA, not applicable.

### Familial Risk of Syncope

The Kaplan-Meier curves (Figure) display the syncope-free survival stratified by presence or absence of relatives (twins, siblings, half-siblings, and cousins) affected by syncope. The syncope-free survival was significantly different among all studied relative pairs, with χ^2^_1_ *df* (χ^2^ = 9.38; *P* = .002) for twins, (χ^2^ = 244.62; *P* < .001) for siblings, (χ^2^ = 29.25; *P* < .001) for half-siblings, (χ^2^ = 35.50; *P* < .001) for cousins, and the separation grade of the syncope-free survival curves was related to the genetic resemblance

**Figure.**
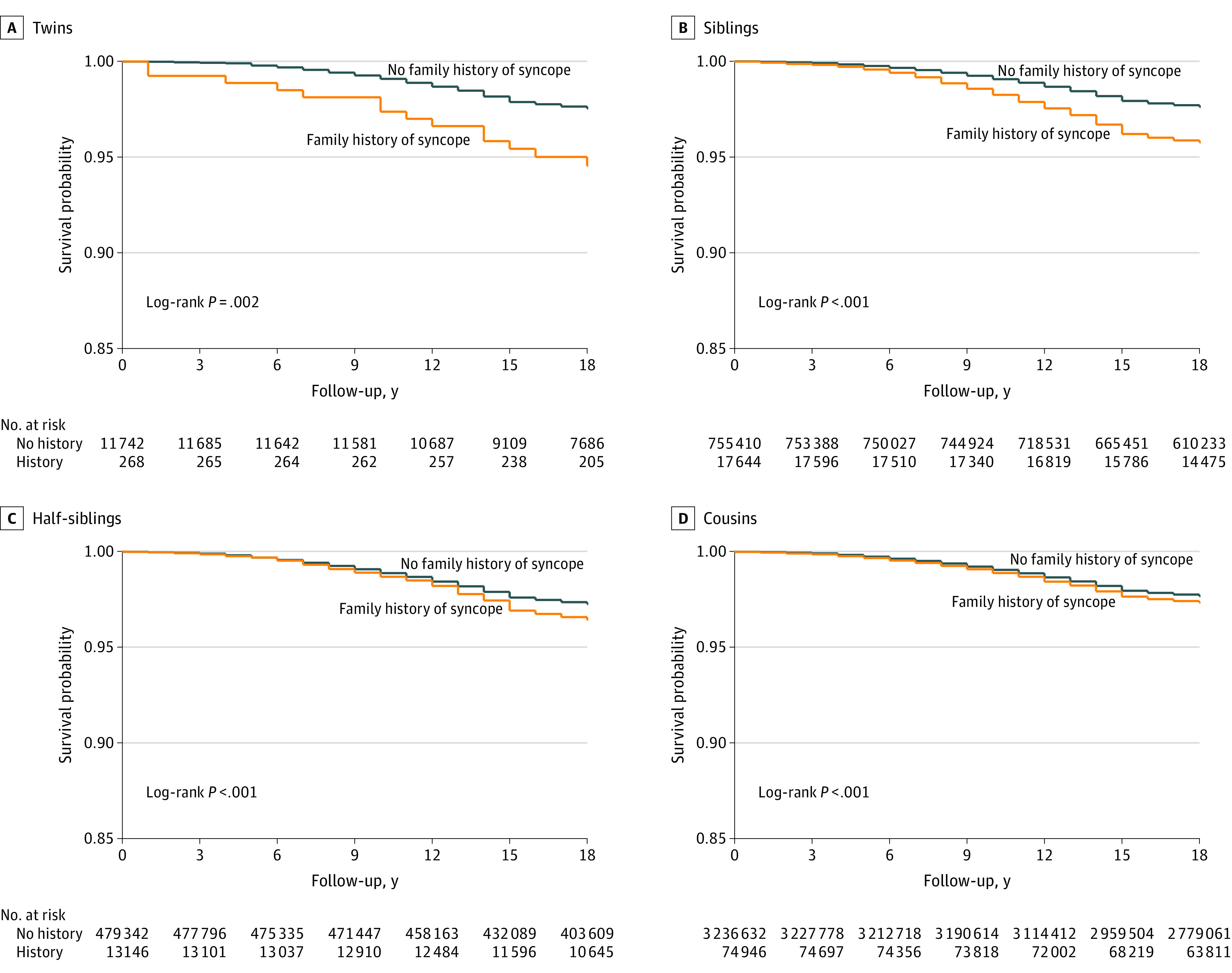
Kaplan-Meier Curves for Syncope-Free Survival According to the Degree of Family Relationship and Documented History of Syncope Among Relatives The start of follow-up period was set to the year 1997 if the participant was born before this date, or to the year of birth if the participant was born after 1997. The end of the follow-up period was set to the year 2015.

The tetrachoric correlations were associated with familial relationships and were *r* = 0.17 (SE, 0.04) among twins, *r* = 0.11 (SE, 0.01) among siblings, *r* = 0.05 (SE, 0.01) among half-siblings, and *r* = 0.02 (SE, 0.00) among cousins. The OR (95% CI) for syncope was 2.39 (1.61-3.53) in twins, 1.81 (1.71-1.91) in siblings, 1.28 (1.20-1.37) in half-siblings, and 1.13 (1.10-1.17) in cousins of those diagnosed with syncope (Table 2).

**Table 2.** Risk of Syncope in Family Members Stratified by the Degree of Relationship and Documented History of Syncope Among Relatives

Variable	Person-years, No.	Cases, No./persons at risk, No.	Incidence rate, cases/ 1000 person-years	Incidence rate ratio (95% CI)	OR (95% CI)^a^		Tetrachoric correlation (SE)^a^
					Model 1	Model 2	
Risk of syncope with affected twins	NA	NA	NA	NA	NA	NA	0.17 (0.04)
Twin not affected^b^	191 532	254/11 739	1.34 (1.18-1.52)	1 [Reference]	1 [Reference]	1 [Reference]	NA
Twin affected	4527	14/271	3.09 (1.83-5.22)	2.31 (1.35-3.95)	2.46 (1.67-3.64)	2.39 (1.61-3.53)	
Risk of syncope with affected sibling	NA	NA	NA	NA	NA	NA	0.11 (0.01)
Sibling not affected^b^	12 885 758	16 921/755 168	1.33 (1.31-1.35)	1 [Reference]	1 [Reference]	1 [Reference]	NA
Sibling affected	302 075	723/17 886	2.39 (2.23-2.57)	1.80 (1.67-1.94)	1.84 (1.74-1.94)	1.81 (1.71-1.91)	
Risk of syncope with affected half-sibling^c^	NA	NA	NA	NA	NA	NA	0.05 (0.01)
Half-sibling not affected^b^	8 237 135	12 701/479 408	1.53 (1.51-1.56)	1 [Reference]	1 [Reference]	1 [Reference]	NA
Half-sibling affected	224 009	445/13 080	1.99 (1.81-2.18)	1.30 (1.18-1.42)	1.30 (1.21-1.39)	1.28 (1.20-1.37)	
Risk of syncope with affected cousin^c^	NA	NA	NA	NA	NA	NA	0.02 (0.00)
Cousin not affected^b^	55 950 037	73 011/326 608	1.31 (1.30-1.32)	1 [Reference]	1 [Reference]	1 [Reference]	NA
Cousin affected	1 292 858	1935/74 970	1.50 (1.43-1.56)	1.15 (1.10-1.20)	1.15 (1.11-1.19)	1.13 (1.10-1.17)	

Abbreviations: NA, not applicable; OR, odds ratio.

^a^ The ORs and tetrachoric correlation were derived from double entry. Model 1 is a crude model (univariate). Model 2 is an adjusted model (multivariate), with adjustments for birth year, sex, county, and educational attainment.

^b^ Reference group.

^c^ The same person can be included in more than 1 family relation.

### Genetic Resemblance and Familial Risk

There was an association between genetic resemblance and tetrachoric correlation and ORs (eFigure 1, eFigure 2, eTable 2, and eTable 3 in the Supplement). The same aforementioned logistic regression analysis was also conducted but with all relative pairs in a single data set with inclusion of an interaction term between the genetic resemblance and family history of syncope. The interaction term was significant (OR = 1.85; 95% CI, 1.71-2.01; *P* for interaction < .001) indicating an association between genetic resemblance and familial ORs of syncope.

### Age and Sex Stratification

Among all familial relationships, the familial ORs were highest at young age (eTable 4, eTable 5, eTable 6, and eTable 7 in the Supplement). Among twins, the highest ORs were observed between male twins, with an adjusted OR of 5.03 (95% CI, 2.57-9.85) (eTable 4 in the Supplement). Among female twins, the adjusted OR was 2.13 (95% CI, 1.17-3.88). Among twins of opposite sex, the ORs were lower. No major sex differences were observed among siblings (eTable 5 in the Supplement). Among half-siblings, no sex differences were observed between male and female pairs, whereas pairs with opposite sex had lower ORs (eTable 6 in the Supplement). Among cousins, no significant sex differences were observed (eTable 7 in the Supplement). Female sex was associated with higher syncope probability (eTable 8 in the Supplement). Associations were also observed with younger age and higher education level. A majority of patients had only 1 syncope registered (twins, 90%; siblings, 80%; half-siblings, 79%; and cousins, 77%) (eTable 9 in the Supplement).

### Sensitivity Analysis

Only 1283 (2.07%) of the 61 861 unique individuals with a diagnosis of syncope had a differential concurrent diagnosis (eTable 1 in the Supplement). Exclusion of families with specific nonreflex syncope TLOC diagnoses did not result in substantial changes in syncope risk (eTable 10 and eTable 11 in the Supplement). A sensitivity test comparing single- and double-entry rendered almost identical results. In double-entry analysis, the confidence intervals were slightly narrower (eTable 12 in the Supplement).

## Discussion

In this cohort study, the results suggest that there may be hereditary components of reflex syncope susceptibility. The strongest aggregation of syncope incidence was observed in twins and pairs of siblings, whereas this association, although significant, was distinctly attenuated in half-siblings and cousin pairs. The familial risk factors and associations correlated with genetic resemblance, suggesting that genetic factors are of importance in the familial aggregation of syncope. The higher risk in young individuals also suggests a genetic basis. Moreover, women demonstrated higher syncope incidence than men across all different levels of family relationships.

The systematic approach to identification of genetic determinants in complex diseases should logically involve studying family aggregation of phenotype and the pattern of aggregation.^21^ The family relationship studies offer unique possibility to explore the associations of genetic and nongenetic familial factors by observing first-degree, second-degree, and third-degree relatives and occurrence of specific phenotypes. First-degree relatives share 50% of their genes, in addition to environmental exposures common to their family. Second-degree relatives (eg, half-siblings) share 25% of their genes, and third-degree relatives (eg, first cousins) share 12.5% of their genes.^19^ In this study, the increasing probability of syncope among relatives of affected individuals followed the pattern of increasing grade of relationship, being highest in twins. Moreover, third degree-relatives usually do not share a household environment. Thus, our observations support the idea of reflex syncope heredity, although the underlying genetical mechanisms appear to be complex.

Studies on reflex syncope have been hampered by the fact that reflex triggers are heterogeneous, orthostatic, visceral, nociceptive, emotional, and situational, to name those most prevalent.^1^ Moreover, the episodic and transient nature of reflex syncope and absence of structural organ changes do not allow well-conducted clinical explorative studies. Instead, researchers are forced to rely on detailed patient’s history and provocation tests such as tilt testing,^1^ with all their limitations.^22^ Notably, the specific diagnostic coding for reflex syncope is lacking, and as a proxy of reflex syncope, the general code of nonspecified syncope (*ICD-10* code R559) has been widely used.^4^ Although this may introduce imprecision, as soon as the specific and definitive cause of the syncopal event has been established, typically related to cardiac or dysautonomic (orthostatic hypotension) phenomena,^1^ the code of nonspecified syncope is no longer used. As reflex syncope is the predominant form of TLOC, representing between 60% to 70% of all syncopal events, especially at younger age,^3^ so the diagnosis of nonspecified syncope can be used as the equivalent of reflex syncope. This assumption was supported by our results after exclusion of families with concurrent definite nonreflex syncope or TLOC diagnoses. Furthermore, the presentation to a doctor is frequently not made in the case of syncope with a benign course; thus, the use of hospital and primary care data inevitably underestimates the real syncope incidence in a population.^6^ It is generally accepted that only between 5% and 10% of all patients experiencing syncope seek medical consultation,^3^ typically when the outcome was alarming or events recur.^1^ The syncope incidence of 2.3% found in the nationwide health care registers is in agreement with the survey-based epidemiological data reporting total lifetime syncope incidence of 20% to 35%^23,24^ (ie, 10 to 15 times higher than the proportion of Swedish patients diagnosed with syncope by different health care practitioners). The overall self-reported incidence of syncopal episode has been estimated to be 18 to 40 cases per 1000 person-years but only 3.6 cases per 1000 person-years are referred for specialist evaluation, which is approximately 10% of the total syncope episodes, whereas approximately 0.7 to 1.8 cases per 1000 person-years are evaluated at emergency departments (5% of all episodes).^3^ The female-male proportion observed in this study is in agreement with previous population-based surveys and confirms higher vasovagal reflex susceptibility among young women.^13,23^ Syncope risk association with higher education level may reflect higher awareness of risks associated with syncope and inclination to report unusual symptoms to health care practitioners. A relatively low proportion (approximately 2%) of syncope diagnoses (*ICD-10* code R559) accompanied by specific diagnostic codes such as orthostatic hypotension or cardiac arrhythmia may be associated with the physicians’ preference of using specific diagnoses for health care registers. This might be because of reimbursement regulations or specific diagnostic codes being more accurate and therapy-oriented. Thus, when the definitive syncope mechanism has been established, a specific disease code may replace the nonspecific *ICD-10* code R559. In contrast, reflex syncope lacks a specific *ICD-10* code and typically dominates the nonspecific group.

Considering the fact that reflex syncope affects only a subset of the population, the propensity for vasovagal reflex seems to be a constant trait in affected individuals throughout their lifetime, and that syncope has characteristic triggers and course suggesting genetic predisposition.^14^ This study suggests that reflex syncope may be heredity, but next steps, such as complex segregation analysis, linkage analysis, and genetic association studies are necessary to identify which type of hereditary factors are involved.^21^ Identifying the crucial genome components potentially responsible for the observed familial syncope aggregation may require both candidate-gene and genome-wide association studies performed in a case-control manner on larger population samples.

### Strengths and Limitations

The large size of the present study is a strength. Another important strength is the use of validated national hospital discharge data,^25^ which allows for the elimination of recall bias. Furthermore, Swedish registers such as the national statistical register and the Swedish Hospital Discharge Register have typically high coverage and high validity of data.^18^

This study has some important limitations that should be mentioned. It is well understood that vasovagal syncope is very common with some estimates being at greater than 40% of the population.^1,6^ The data collected here is numerically large, but the incidence of syncope is substantially lower than anticipated by syncope physicians. This may be explained by the fact that many patients do not seek medical advice. However, we believe this is a nondifferential bias with regards to the estimated familial associations. Another limitation is that the *ICD-10* R559 code could not be specifically validated. On the other hand, the female-male proportion and the young age at onset suggest that the *ICD-10* code is valid for reflex syncope. We did not assess the effect of consanguinity on our results. However, the proportion of consanguinity in our data set was 0.2% and we considered it unlikely that consanguinity may have affected the results.

## Conclusions

This study’s results suggest that risk of syncope among relatives of individuals with syncope is associated with the degree of relationship, being strongest in twins and siblings but still significant in third-degree relatives. This suggests genetic components of reflex syncope susceptibility. This study’s findings also suggest that women are more prone to syncope independently of family relationship and other sex-related genetic factors may be involved.

## Notes

Supplement. **eTable 1.** Definite Causes of Syncope/Transient Loss of Consciousness Applied for Sensitivity Analysis **eTable 2.** Correlation Between Genetic Resemblance and Tetrachoric Correlation **eTable 3.** Correlation Between Genetic Resemblance and Odds **eTable 4.** Stratified Tetrachoric Correlations and Odds Ratios (OR) According to Sex and Age Among Twins **eTable 5.** Stratified Tetrachoric Correlations and Odds Ratios (OR) According to Sex and Age Among Siblings **eTable 6.** Stratified Tetrachoric Correlations and Odds Ratios (OR) According to Sex and Age Among Half-Siblings **eTable 7.** Stratified Tetrachoric Correlations and Odds Ratios (OR) According to Sex and Age Among Cousins **eTable 8.** Other Risk Factors for Syncope **eTable 9.** Number of Registrations for Syncope (Unique Individuals) **eTable 10.** Number of Families With Syncope Before and After Exclusion of Families With Other Causes of Syncope **eTable 11.** Sensitivity Analysis With Exclusion of Individuals and Families With Other Causes of Syncope **eTable 12.** Risk of Syncope Among Different-Degree Relatives if One Relative is Affected vs Unaffected by Syncope in Two Models: Using Single- and Double-Entry of Relative Pairs **eFigure 1.** Tetrachoric Correlations vs Genetic Resemblance for Twins, Siblings, Half-Siblings, and Cousins **eFigure 2.** Odds Ratios vs Genetic Resemblance for Twins, Siblings, Half-Siblings, and Cousins
