# Supplementary material for: Risk Factors for Syncope Associated With Multigenerational Relatives With a History of Syncope
Source: JAMA Netw Open. 2021 Mar 30;4(3):e212521. doi: 10.1001/jamanetworkopen.2021.2521 (PMC8010588; doi:10.1001/jamanetworkopen.2021.2521)
Supplement: Supplement. — eTable 1. Definite Causes of Syncope/Transient Loss of Consciousness Applied for Sensitivity Analysis eTable 2. Correlation Between Genetic Resemblance and Tetrachoric Correlation eTable 3. Correlation Between Genetic Resemblance and Odds eTable 4. Stratified Tetrachoric Correlations and Odds Ratios (OR) According to Sex and Age Among Twins eTable 5. Stratified Tetrachoric Correlations and Odds Ratios (OR) According to Sex and Age Among Siblings eTable 6. Stratified Tetrachoric Correlations and Odds Ratios (OR) According to Sex and Age Among Half-Siblings eTable 7. Stratified Tetrachoric Correlations and Odds Ratios (OR) According to Sex and Age Among Cousins eTable 8. Other Risk Factors for Syncope eTable 9. Number of Registrations for Syncope (Unique Individuals) eTable 10. Number of Families With Syncope Before and After Exclusion of Families With Other Causes of Syncope eTable 11. Sensitivity Analysis With Exclusion of Individuals and Families With Other Causes of Syncope eTable 12. Risk of Syncope Among Different-Degree Relatives if One Relative is Affected vs Unaffected by Syncope in Two Models: Using Single- and Double-Entry of Relative Pairs eFigure 1. Tetrachoric Correlations vs Genetic Resemblance for Twins, Siblings, Half-Siblings, and Cousins eFigure 2. Odds Ratios vs Genetic Resemblance for Twins, Siblings, Half-Siblings, and Cousins [file jamanetwopen-e212521-s001.pdf]

Supplemental Online Content

Fedorowski A, Pirouzifard M, Sundquist J, Sundquist K, Sutton R, Zöller B. Risk factors for syncope associated with multigenerational relatives with a history of syncope. *JAMA Netw Open*. 2021;4(3):e212521. doi:10.1001/jamanetworkopen.2021.2521

eTable 1. Definite Causes of Syncope/Transient Loss of Consciousness Applied for Sensitivity Analysis
eTable 2. Correlation Between Genetic Resemblance and Tetrachoric Correlation
eTable 3. Correlation Between Genetic Resemblance and Odds
eTable 4. Stratified Tetrachoric Correlations and Odds Ratios (OR) According to Sex and Age Among Twins
eTable 5. Stratified Tetrachoric Correlations and Odds Ratios (OR) According to Sex and Age Among Siblings
eTable 6. Stratified Tetrachoric Correlations and Odds Ratios (OR) According to Sex and Age Among Half-Siblings
eTable 7. Stratified Tetrachoric Correlations and Odds Ratios (OR) According to Sex and Age Among Cousins
eTable 8. Other Risk Factors for Syncope
eTable 9. Number of Registrations for Syncope (Unique Individuals)
eTable 10. Number of Families With Syncope Before and After Exclusion of Families With Other Causes of Syncope
eTable 11. Sensitivity Analysis With Exclusion of Individuals and Families With Other Causes of Syncope
eTable 12. Risk of Syncope Among Different-Degree Relatives if One Relative is Affected vs Unaffected by Syncope in Two Models: Using Single- and Double-Entry of Relative Pairs
eFigure 1. Tetrachoric Correlations vs Genetic Resemblance for Twins, Siblings, Half-Siblings, and Cousins
eFigure 2. Odds Ratios vs Genetic Resemblance for Twins, Siblings, Half-Siblings, and Cousins

This supplemental material has been provided by the authors to give readers additional information about their work.

© 2021 Fedorowski A et al. *JAMA Network Open.*

**eTable 1.** Definite Causes of Syncope/Transient Loss of Consciousness Applied for Sensitivity Analysis	
**Cause of syncope**	ICD-10
**Orthostatic hypotension**	I951
**Cardiac syncope:** structural heart disease, bradyarrhythmias, and tachyarrhythmias	
Coronary heart disease (CHD)	I20-I25
Aortic stenosis	I350
Cardiomyopathy	I42
Heart failure	I50
Aortic dissection	I710
Pulmonary embolism	I26
Primary pulmonary hypertension	I270
Acute myocarditis	I40
AV block II and III	I441, I442
Tachycardia paroxysmalis	I47
Atrial fibrillation	I48
Sick-sinus syndrome	I495
Myxoma	D219F
**Neurological causes**	
Parkinson’s disease	G20
Stroke	I60, I61, I62, I63, I64
TIA	G45
Migraine	G43
Epilepsy	G40, G41
**Other causes**	
Chronic adrenal insufficiency	E271, E272, E273, E274
Hypopituitarism	E23

© 2021 Fedorowski A et al. *JAMA Network Open.*

eTable 2. Correlation Between Genetic Resemblance and Tetrachoric Correlation		
R[*]	P value	R^{2**}
0.994	0.006	0.988

[*] Correlation coefficient; [**]the coefficient of determination.

eTable 3. Correlation Between Genetic Resemblance and Odds Ratios		
R[*]	P value	R^{2**}
0.984	0.016	0.968

[*] Correlation coefficient; [**]the coefficient of determination.

eTable 4. Stratified Tetrachoric Correlations and Odds Ratios (OR) According to Sex and Age Among Twins

	Tetrachoric correlation (SE)	OR (95%CI)	
		Crude	Adjusted*
Stratified: Sex			
Male -Male	0.31 (0.07)	5.39 (2.76-10.53)	5.03 (2.57-9.85)
Female – Female	0.15 (0.06)	2.17 (1.19-3.95)	2.13 (1.17-3.88)
Male – Female	0.07 (0.11)	1.52 (0.47-4.90)	1.51 (0.47-4.89)
Female -Male	0.07 (0.11)	1.52 (0.47-4.90)	1.53 (0.47-4.93)
Stratified: Age at end of study			
Age <= 20	0.24 (0.06)	3.66 (2.00-6.68)	2.72 (1.48-5.00)
20 < Age <= 30	0.16 (0.07)	2.26 (1.24-4.12)	2.18 (1.19-4.00)
Age > 30	0.04 (0.09)	1.25 (0.46-3.41)	1.25 (0.46-3.43)

© 2021 Fedorowski A et al. *JAMA Network Open.*

**eTable 5.** Stratified Tetrachoric Correlations and Odds Ratios (OR) According to Sex and Age Among Siblings			
	Tetrachoric correlation (SE)	OR (95%CI)	
		Crude	Adjusted*
Stratified: Sex			
Male -Male	0.10 (0.01)	1.83 (1.58-2.11)	1.81 (1.57-2.10)
Female – Female	0.13 (0.01)	1.96 (1.80-2.15)	1.87 (171-2.04)
Male – Female	0.10 (0.01)	1.79 (1.60-1.99)	1.78 (1.60-1.99)
Female -Male	0.10 (0.01)	1.79 (1.60-1.99)	1.77 (1.59-1.98)
Stratified: Age at end of study			
Age <= 24	0.12 (0.01)	1.91 (1.76-2.08)	1.81 (1.67-1.97)
24 < Age <= 39	0.11 (0.01)	1.78 (1.63-1.94)	1.70 (1.56-1.85)
Age > 39	0.09 (0.01)	1.66 (1.46-1.87)	1.64 (1.45-1.85)

**eTable 6.** Stratified Tetrachoric Correlations and Odds Ratios (OR) According to Sex and Age Among Half-Siblings			
	Tetrachoric correlation (SE)	OR (95%CI)	
		Crude	Adjusted*
Stratified: Sex			
Male -Male	0.07 (0.02)	1.47 (1.24-1.75)	1.45 (1.22-1.73)
Female – Female	0.07 (0.02)	1.40 (1.26-1.55)	1.37 (1.24-1.52)
Male – Female	0.02 (0.01)	1.15 (0.99-1.34)	1.15 (0.99-1.34)
Female -Male	0.02 (0.01)	1.52 (0.99-1.34)	1.15 (0.99-1.34)
Stratified: Age at end of study			
Age <= 24	0.06 (0.01)	1.41 (1.23-1.58)	1.42 (1.26-1.59)
24 < Age <= 39	0.04 (0.01)	1.27 (1.14-1.40)	1.26 (1.14-1.40)
Age > 39	0.03 (0.01)	1.16 (1.00-1.35)	1.15 (0.99-1.34)

© 2021 Fedorowski A et al. *JAMA Network Open.*

**eTable 7.** Stratified Tetrachoric Correlations and Odds Ratios (OR) According to Sex and Age Among Cousins			
	Tetrachoric correlation (SE)	OR (95%CI)	
		Crude	Adjusted*
Stratified: Sex			
Male -Male	0.01 (0.007)	1.09 (1.00-1.19)	1.09 (1.00-1.18)
Female – Female	0.03 (0.005)	1.19 (1.13-1.26)	1.15 (1.09-1.21)
Male – Female	0.02 (0.006)	1.13 (1.06-1.21)	1.13 (1.06-1.21)
Female -Male	0.02 (0.006)	1.13 (1.06-1.21)	1.13 (1.06-1.21)
Stratified: Age at end of study			
Age <= 24	0.04 (0.01)	1.21 (1.15-1.28)	1.20 (1.13-1.27)
24 < Age <= 39	0.02 (0.004)	1.10 (1.05-1.16)	1.09 (1.03-1.14)
Age > 39	0.01 (0.001)	1.09 (1.02-1.16)	1.08 (1.01-1.16)

**eTable 8.** Other Risk Factors for Syncope (Odds ratios determined with logistic regression for syncope in family members stratified by the degree of relationship.)					
Risk factors	Reference	Twins	Siblings	Half-siblings	Cousin
History of syncope among relatives	No history	2.39 (1.61 – 3.53)	1.81 (1.71 – 1.91)	1.28 (1.20 – 1.37)	1.13 (1.10 – 1.17)
Year of birth	Per year	1.00 (0.99 – 1.01)	1.01 (1.01 – 1.01)	1.01 (1.01 – 1.01)	1.01 (1.01 – 1.01)
Sex	Male	1.80 (1.51 – 2.15)	1.73 (1.69 – 1.77)	1.87 (1.82 – 1.92)	1.72 (1.70 – 1.73)
Level of education (per education category)*	Unknown education/ children	1.13 (1.01 – 1.26)	1.08 (1.06 – 1.09)	1.02 (1.01 – 1.04)	1.04 (1.04 – 1.05)
*Unknown education/children=1; elementary school=2; high school=3; college/university=4<br>Adjusted for year of birth, sex, level of education, and county of residence.					

© 2021 Fedorowski A et al. *JAMA Network Open.*

eTable 9. Number of Registrations for Syncope (Unique Individuals)

Number	Twins	Siblings	Half-Siblings	Cousins
1	433 (80.33)	28528 (80.29)	5198 (79.08)	18446 (77.69)
2	76 (14.10)	4649 (13.08)	876 (13.33)	3358 (14.14)
3	20 (3.71)	1223 (3.44)	243 (3.70)	1013 (4.27)
>3	10 (1.86)	1130 (3.18)	256 (3.89)	927 (3.90)

© 2021 Fedorowski A et al. *JAMA Network Open.*

**eTable 10.** Number of Families With Syncope Before and After Exclusion of Families With Other Causes of Syncope		
**Relatives**	Number of families before exclusion	Number of families that were excluded (%)
**Twins**	12010	8 (0.07)
**Siblings**	773054	705 (0.09)
**Half-Siblings**	140611	299 (0.21)
**Cousins**	404511	896 (0.22)

**eTable 11.** Sensitivity Analysis With Exclusion of Individuals and Families With Other Causes of Syncope (Tetrachoric correlation and odds ratios (OR) after exclusion.)			
**Relatives**	Tetrachoric correlation (SE)	OR (95%CI)	
		Crude	Adjusted*
**Twins**	0.17 (0.04)	2.94 (1.63-5.23)	2.46 (1.66-3.64)
**Siblings**	0.11 (0.01)	1.84 (1.74-1.95)	1.81 (1.71-1.91)
**Half siblings**	0.05 (0.01)	1.28 (1.20-1.38)	1.27 (1.19-1.36)
**Cousins**	0.02 (0.003)	1.15 (1.11-1.9)	1.13 (1.09-1.17)

© 2021 Fedorowski A et al. *JAMA Network Open.*

eTable 12.

Risk of Syncope Among Different-Degree Relatives if One Relative is Affected vs Unaffected by Syncope in Two Models: Using Single- and Double-Entry of Relative Pairs

Twins	Crude model	Adjusted model*
	OR (95% CI)	OR (95% CI)
Single entry	2.46 (1.42-4.28)	2.56 (1.47-4.46)
Double entry	2.46 (1.67-3.64)	2.39 (1.61-3.53)

Siblings	Crude model	Adjusted model*
	OR (95% CI)	OR (95% CI)
Single entry	1.84 (1.70-1.98)	1.81 (1.68-1.95)
Double entry	1.84 (1.74-1.94)	1.81 (1.71-1.91)

Half-Siblings	Crude model	Adjusted model*
	OR (95% CI)	OR (95% CI)
Single entry	1.30 (1.18-1.43)	1.28 (1.17-1.41)
Double entry	1.30 (1.21-1.39)	1.28 (1.20-1.37)

Cousins	Crude model	Adjusted model*
	OR (95% CI)	OR (95% CI)
Single entry	1.15 (1.10-1.20)	1.13 (1.08-1.18)
Double entry	1.15 (1.11-1.19)	1.13 (1.10-1.17)

OR, odds ratio. CI, confidence interval. *Adjusted for sex, level of education, county and year of birth.

© 2021 Fedorowski A et al. *JAMA Network Open.*

eFigure 1. Tetrachoric Correlations vs Genetic Resemblance for Twins, Siblings, Half-Siblings, and Cousins

X axis is genetic resemblance i.e. 66% (=0.66) for twins (according to Weinberg's differential method), 50% (=0.5) for siblings, 25% for half siblings and 12.5% for cousins. Y axis is tetrachoric correlations.

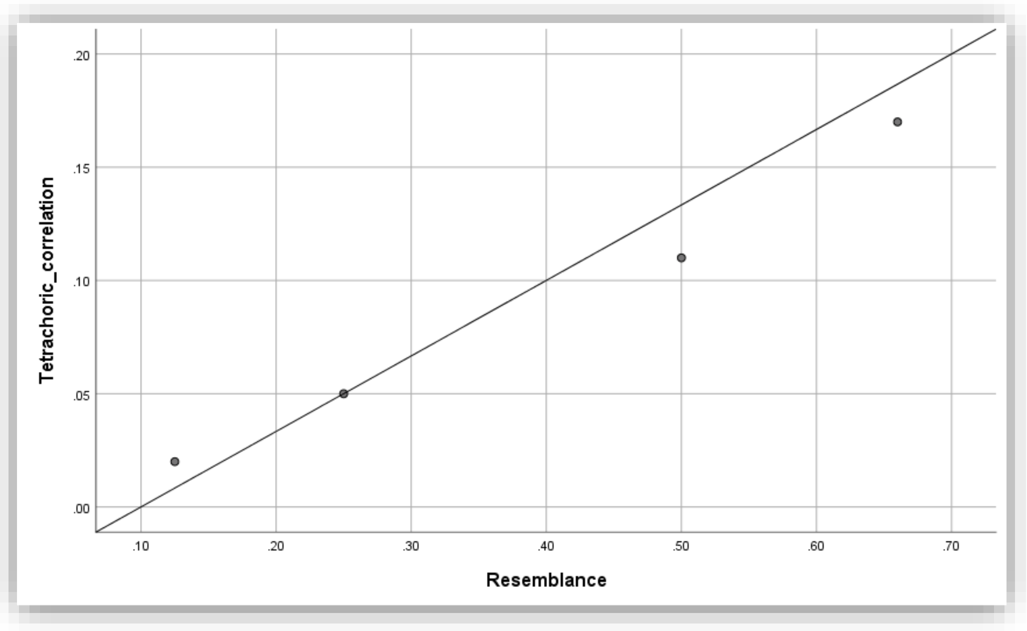

eFigure 2. Odds Ratios vs Genetic Resemblance for Twins, Siblings, Half-Siblings, and Cousins

X axis is genetic resemblance i.e. 66% (=0.66) for twins (according to Weinberg's differential method), 50% (=0.5) for siblings, 25% for half siblings and 12.5% for cousins. Y axis is odds ratios.

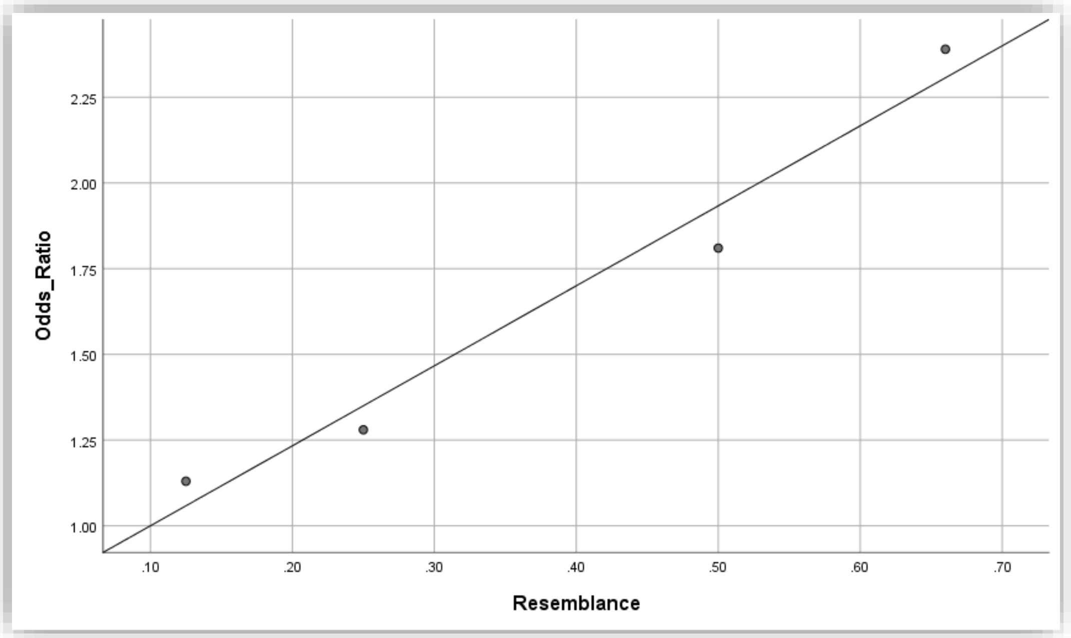

© 2021 Fedorowski A et al. *JAMA Network Open.*
